# Common Psychological Factors in Chronic Diseases

**DOI:** 10.3389/fpsyg.2019.02727

**Published:** 2019-12-06

**Authors:** Ciro Conversano

**Affiliations:** Department of Surgical, Medical, Molecular and Critical Pathology, University of Pisa, Pisa, Italy

**Keywords:** psychological factors, quality of life, emotional distress, chronic diseases, adherence

The construct of “*chronic physical diseases*” (CPDs) encompasses a number of heterogeneous conditions that have persisting lifelong effects on the quality of life (QoL) and subjective well-being (Sprangers et al., 2000). According to epidemiological studies, CPDs are constantly increasing, not only in Western countries but also in developing/emerging countries, with certain prevalent differences regarding CPD type (Vos et al., 2016), raising questions on the multifactorial genesis of this phenomenon. The role of psychiatric disorders is, for example, well-known as comorbid conditions able to affect the course of CPD with a number of *sequelae* and complications (Daré et al., 2019).

The most common CPDs (namely, cardiovascular disease, diabetes mellitus, neoplastic diseases, asthma, arthritis, and osteoporosis) are often complicated by psychiatric symptoms or emotional/psychological subjective suffering (Martino G. et al., 2019; Rosa et al., 2019), a datum that underlines the close correlation that exists between such conditions. However, the relationships and the mutual influences between CPD and psychopathological manifestations are far from established (Marchini et al., 2018; Miniati et al., 2018; Martino G. et al., 2019).

Findings on psychological/psychopathological dimensions in patients with CPD, both from a cross-sectional and from a lifetime perspective, are available in the literature, with an emphasis on their impact on cognitive functioning, emotional processing, exposition to stressful events (SLEs) and adversities, medical and psychological outcomes, and combined interventions and therapies (Bernard et al., 2018; McGilton et al., 2018; Shao et al., 2019). A number of studies have, for instance, already explored the impact of signs and symptoms belonging to the realm of psychopathological disorders on the most common CPDs, with a measure of the subjective perception of well-being and QoL (Megari, 2013). More specifically, alexithymia, anxiety, depression, psychological distress, sleep quality, and emotional dysregulations have been systematically assessed in patients with fibromyalgia, Type 2 diabetes, psoriasis, and osteoporosis (Palagini et al., 2016; Catalano et al., 2018; Martino et al., 2018a,b; Cristina et al., 2019; Kelly et al., 2019; Marchi et al., 2019; Martino M. L. et al., 2019; Settineri et al., 2019a,b). This datum represents the increasing tendency of the scientific community to take an interest in the aforementioned connection between the psychological and physical spheres, hypothesizing a positive correlation between the two, where a higher psychological and QoL malaise correspond to the increasing severity of the pathology.

In nearly all of the abovementioned conditions, cognitive functioning and performances were impaired, as enhanced by studies with cognitive tasks, again raising questions as to the different weight and role of metabolic dysregulations vs. comorbid anxiety or depressive disorders in determining the severity of cognitive dysfunctions (Guicciardi et al., 2019). For example, emotional processing and depression has been found to enhance “*pain catastrophization*,” which could be described as the cognitive attitude of interpreting the experience of pain in an excessively negative manner, during upper endoscopy in young, especially female, patients, when exposed for the first time to diagnostic procedures and pain therapies (Sullivan et al., 2001; Lauriola et al., 2019). A number of studies also highlight the reciprocal influences between psychological and medical conditions in affecting cognitive performances and emotional reactions among children and young adults, with relevant *sequelae* in adulthood and in the elderly, while other studies have opened up debate on the interaction of age, gender, and medical conditions on mental status (see the association between early childhood SLEs, depression, cognitive functioning, and lipids' metabolism alterations; Stewart et al., 2000; Péterfalvi et al., 2019). Other studies demonstrate how an early diagnosis of a neuropsychiatric condition (such as ADHD) may change the electrophysiological characteristics and the overall subjective neuropsychological profile during adulthood (Angela et al., 2018; Klein et al., 2019), as possibly determined by the occurrence of manic symptoms and PTSD in young adults (Dell'Osso et al., 2014) or the emotional suppression or oneiric perturbation in subjects affected by psychosomatic illnesses (Settineri et al., 2019a,b).

Overall, these studies demonstrate the importance of a multidisciplinary approach in treating patients affected by CPD and both psychological and psychopathological disorders. Both psychological and physical interventions in patients with CPD could ameliorate prognosis, considering the described relationships between psychological factors and CPD, as identified by studies on the positive impact of a healthy psychological functioning on CPD (Schiavon et al., 2017; Gentili et al., 2019). Psycho-educational interventions, mindfulness-based cognitive therapy, non-invasive brain stimulation techniques, peer-to-peer supports, and a health-based approach have been all tested with promising results in patients with CPD (Castelnuovo et al., 2015; De Jong et al., 2016; Naro et al., 2016; Callus and Pravettoni, 2018; Conversano et al., 2019).

In conclusion, it could be inferred that the bidirectional association between CPD and psychopathological factors might lead to an exacerbation of both conditions, with mechanisms that are only partially known and described. However, a relevant corpus of knowledge supports the need for an integrated approach (physical, psychological, and psychopathological) that takes into account the subjective experience of the single patient from a lifetime perspective. As a consequence, it is necessary to consider the corollary of symptoms that the patient who suffers from a chronic disorder manifests as a unitary corpus, where it is possible to intervene both with medical and psychological science to improve QoL and therefore physical symptoms. In the history of the patient's illness, the weight of psychological variables plays a fundamental and non-negligible role when the doctor's interest is that of treating the patient from a long-term perspective.

The development of therapeutic interventions able to fuse different perspectives into a tailor-made interdisciplinary management approach in a single patient and the development of a quality body of research on the topic are future challenges in order to improve QoL and the subjective well-being of patients with CPD and psychopathological signs and symptoms.

## Author Contributions

CC was responsible for writing the entire opinion article, for checking the adequacy of references and of all aspects of layout.

### Conflict of Interest

The author declares that the research was conducted in the absence of any commercial or financial relationships that could be construed as a potential conflict of interest.
